# Nanocarrier Drug Delivery Systems: Characterization, Limitations, Future Perspectives and Implementation of Artificial Intelligence

**DOI:** 10.3390/pharmaceutics14040883

**Published:** 2022-04-18

**Authors:** Samar Zuhair Alshawwa, Abeer Ahmed Kassem, Ragwa Mohamed Farid, Shaimaa Khamis Mostafa, Gihan Salah Labib

**Affiliations:** 1 Department of Pharmaceutical Sciences, College of Pharmacy, Princess Nourah bint Abdulrahman University, P.O. Box 84428, Riyadh 11671, Saudi Arabia; samarzuhair@yahoo.com or; 2 Department of Pharmaceutics and Pharmaceutical Technology, Faculty of Pharmacy, Pharos University in Alexandria, Alexandria 21523, Egypt; ragwa.m.f@gmail.com (R.M.F.); jilabib@gmail.com (G.S.L.); 3 Department of Pharmaceutics and Pharmaceutical Technology, Faculty of Pharmacy, Delta University for Science and Technology, Gamasa 11152, Egypt; shaimaa.khamiss@gmail.com

**Keywords:** nanocarriers characterization, challenges, artificial intelligence, future perspectives, stability, regulatory aspects, safety considerations

## Abstract

There has been an increasing demand for the development of nanocarriers targeting multiple diseases with a broad range of properties. Due to their tiny size, giant surface area and feasible targetability, nanocarriers have optimized efficacy, decreased side effects and improved stability over conventional drug dosage forms. There are diverse types of nanocarriers that have been synthesized for drug delivery, including dendrimers, liposomes, solid lipid nanoparticles, polymersomes, polymer–drug conjugates, polymeric nanoparticles, peptide nanoparticles, micelles, nanoemulsions, nanospheres, nanocapsules, nanoshells, carbon nanotubes and gold nanoparticles, etc. Several characterization techniques have been proposed and used over the past few decades to control and predict the behavior of nanocarriers both in vitro and in vivo. In this review, we describe some fundamental in vitro, ex vivo, in situ and in vivo characterization methods for most nanocarriers, emphasizing their advantages and limitations, as well as the safety, regulatory and manufacturing aspects that hinder the transfer of nanocarriers from the laboratory to the clinic. Moreover, integration of artificial intelligence with nanotechnology, as well as the advantages and problems of artificial intelligence in the development and optimization of nanocarriers, are also discussed, along with future perspectives.

## 1. Introduction

Pharmaceutical research seeking to enhance drug bioavailability, increase stability and improve organ targeting has been progressively advanced. Pharmaceutical nanocarriers are drug delivery vehicles of submicron size and high versatility. They include polymeric, lipidic and inorganic nanoparticles, liposomes, nanotubes, nanocomplexes, niosomes and many others. In principle, ligands can be attached to the surface of nanocarriers for better uptake and targetability [1,2,3]. Drugs can be either dispersed into the nanocarrier matrix or located within the nanocarrier layers. Nanocarriers offer several advantages over traditional drug therapies as it is easier to customize their size, charge, surface properties and targeting moieties to regulate their uptake, biodistribution, targeting and elimination [4]. They can be administered via many different routes, e.g., parenteral [5], nasal [6], topical [7,8] or oral routes [9,10]. Owing to the preceding advantages, there is an increasing demand for the development of nanocarriers targeting multiple diseases with a broad range of properties. Hence, several characterization techniques have been proposed and used over the past few decades to control and predict the behavior of nanocarriers both in vitro and in vivo. The commonly employed characterization techniques are used to assess the physicochemical properties, drug loading, release rate, mechanical behavior, stability, tissue permeability, possible toxicity and in vivo fate of nanocarriers. In this review, we summarize the indispensable characterization methods commonly used for most nanocarrier drug delivery systems (DDSs). Moreover, the limitations of these methods are presented, along with the regulatory difficulties and scalability issues confronting the manufacturing of nanocarriers.

## 2. Physicochemical Characterization

Physicochemical properties of nanocarriers include their particle size, particle size distribution, surface charge, hydrophobicity and morphology. The determination of these physicochemical properties for the drug nanocarrier can predict, to a great extent, several aspects, including physical stability and entrapment efficiency [11].

### 2.1. Particle Size and Polydispersity

The most important characteristics of nanocarriers are particle size, shape and dispersity (heterogeneity of particles in terms of size expressed by polydispersity index (PDI)). The particle size and shape affect the biodistribution and elimination of nanocarriers [12,13,14]. They also affect their attachment, firm adhesion [15], phagocytosis [16], circulation half-life, cellular distribution [17], cellular uptake and endocytosis [18,19].

In the following sections, we give an overview of the foremost, routinely used strategies to determine particle size and PDI.

#### 2.1.1. Dynamic Light Scattering Spectroscopy

**Figure 1:**
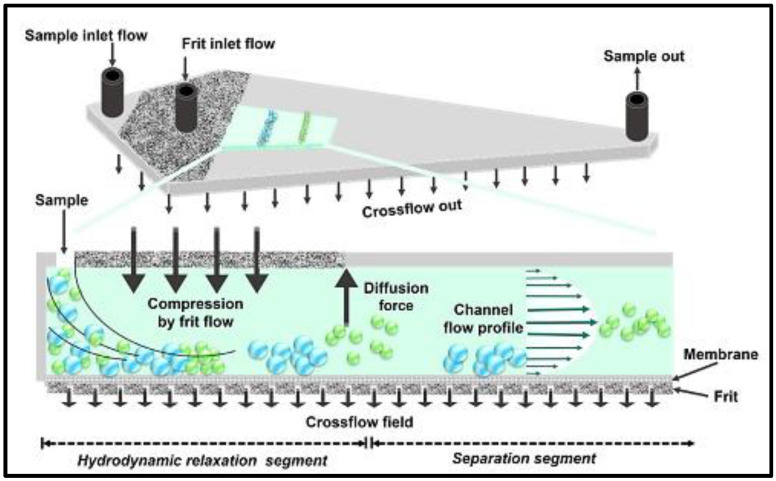
Schematic representation of an asymmetric flow field flow fractionation channel equipped with a frit inlet (FI-AF4). Frit inlet flow propels sample components towards the accumulation wall, allowing their hydrodynamic relaxation without stopping their axial migration. Adapted with permission from [22], Elsevier, 2021.

Dynamic light scattering (DLS) determines the particle diameter with the aid of Brownian motion and light scattering properties. However, large particles may not be discovered by the DLS method since their movement may be too slow [20]. The mean particle diameter and PDI are measured using a particle sizer equipped with particle sizing software [21]. Samples must be in a liquid state, solution or dilute suspension of known viscosity. This technique is sensitive to impurities and can measure particles between 1 nm and 10 μm in diameter. The obtained data of particle size, size distribution and PDI are convenient for statistical analysis. However, some limitations might confine the application of DLS such as the unreliable results of polydisperse and multimodal samples, sedimentation of large particles and sample concentration. These limitations can be overcome by adding a fractionation step to obtain fractions of different particles sizes prior to measurement by the DLS method [22]. The asymmetrical flow field flow fractionation (AF4) method involves the separation of samples into an unpacked, narrow-opened channel [23]. Concisely, a single-carrier flow is withdrawn from the channel inlet that splits into the channel flow and the crossflow, Figure 1. The channel flow exhibits a parabolic velocity profile line that carries nanoparticles to the channel outlet to be detected. On the other hand, the crossflow moves from the top to the bottom of the channel, forcing the nanoparticles to move down on the accumulation wall that is made of an ultrafiltration membrane covered by a porous frit. At the end, the nanoparticles’ diffusion restricts the crossflow field, allowing for size fractionation where smaller particles reach an equilibrium position higher up in the channel with faster channel flow velocity that allows for earlier elution than larger particles [22,23,24]. Moreover, temperature and pH may impact the reliability of the measurements. DLS is generally considered inappropriate for measurements within biological media [25]. One of the most important aspects in a new and specific AF4 method is that it needs to be established for each kind of measured nanoparticles sample depending on its composition, average size, surface properties and size distribution [26]. The results obtained by this method are usually confirmed by scanning electron microscopy (SEM) or transmission electron microscopy (TEM) [22]. Other limitations may comprise the assumption of having spherical samples, which may not be true in all cases, having turbid or translucent samples where light absorption by the dispersed particles can interfere with the detection and having aggregated particles that may not be distinguished from individual particles, and the method requires that the solvent refractive index must be accurately known [27].

#### 2.1.2. Static Light Scattering

Static light scattering depends on measuring the intensity of scattered light waves depending on scattering angle, followed by the application of an acceptable mathematical model (usually Mie theory) to convert the scattering pattern to particle size distribution. This model assumes that the scattered particles are homogeneous, spherical, fail to interact and have a definite refractive index. Actually, most biopolymeric particles do not agree with such assumptions. Moreover, sample preparation necessitates prior dilution and shaking, which might change the integrity or aggregation of biopolymer particles. Accordingly, the results pooled from static light scattering should be used with care [28].

#### 2.1.3. Atomic Force Microscopy

Atomic force microscopy (AEM) allows particle size measurement with ultra-high resolution based on scanning the submicron particle levels with a probe tip of atomic scale. The appliance provides a topographic map of the sample depending on the force between a sharp probe and the surface of the sample. This technique allows the imaging of non-conducting samples without any special treatment, which, in turn, facilitates the imaging of delicate biological and polymeric nanocarriers [29]. Most significantly, it provides the foremost correct description of size and size distribution without applying any algorithmic treatment. However, it is worth mentioning that accurate data collection and interpretation of results requires strong expertise, especially when dealing with complex samples or specimens such as biological cells. The main concerns are those related to the quality of tip and support surface chemistries and the expectation of their possible alteration during data collection of the shape and size of measured vesicles. Other limitations may include the poor sampling techniques and time consumption caused by the slow scanning technique of the instrument, which lacks the capability to detect specific molecules. However, the latter limitation can be resolved by a more advanced single—molecule force spectroscopy with AFM cantilever tip carrying certain ligands or molecules that can detect specific functional groups. Therefore, a deep understanding of the principle and limitations of different AFM modalities is essential before users proceed with their first experiment [27,30].

#### 2.1.4. Centrifugal Liquid Sedimentation

Centrifugal liquid sedimentation (CLS) is a fractionation method in which different monodisperse fractions within a sample are isolated by centrifugation before particle size measurement [31]. Theoretically, CLS is more suitable for polydisperse samples. However, the fractionation becomes greatly complicated if the size distribution is too broad. Spherical nanocarriers with narrower size distribution and densities are better candidates for CLS measurements [32,33]. Samples also must not undergo any changes, chemically or physically, in the suspension during sedimentation. Moreover, the refractive index and densities of the particles and liquid medium must differ from each other to give reliable results. It was previously reported that both DLS and CLS methods were suitable and robust for the determination of particle size of silica nanoparticles suspension in the range of 35–50 nm.

### 2.2. Surface Charge and Hydrophobicity

The surface properties of nanocarriers significantly influence their bioavailability, stability, cellular uptake and biodistribution [12,34,35]. The zeta (ζ) potential, expressing the surface charge, indicates possible electrostatic interactions between the nanocarrier units, affects their aggregation tendencies and helps to select proper coating materials [36]. It can be determined by applying an electrical current through the sample while recording the movement of the nanocarriers using laser Doppler velocimetry [37]. So far, electrophoretic light scattering is the most popular method due to its accuracy, sensitivity and versatility. Overall, measuring zeta potential can be highly sensitive to ionic strength and pH. Sample dilution is usually required before measurement. Mixtures of oppositely charged nanocarriers might affect the reliability of the interpreted data [20].

The hydrophobicity of nanocarriers can be assessed by adsorption probe method, hydrophobic interaction chromatography, contact angle measurements and biphasic partitioning. In addition, X-ray photon correlation spectroscopy helps to identify specific chemical groups on the surface of nanocarriers and eventually predicts their hydrophobicity [38].

### 2.3. Morphology of Nanocarriers

The morphology of nanocarriers and their aggregation behavior are important factors for different biological properties, including their half-life, targeting efficiency and toxicity [39]. Several non-spherical shapes, including discs, ellipsoids, cylinders, hemispheres, cubes, cones and other complex shapes, have a profound effect on those biological processes [12]. On the one hand, atomic force microscopy grants the study of the shape of nanocarriers with high resolution without altering sample properties before measurement. On the other hand, electron microscopy techniques, namely SEM and TEM, offer numerous benefits on morphological and particle sizing characterization but have minimal information on the size distribution and true population mean.

#### 2.3.1. Scanning Electron Microscopy

**Figure 2:**
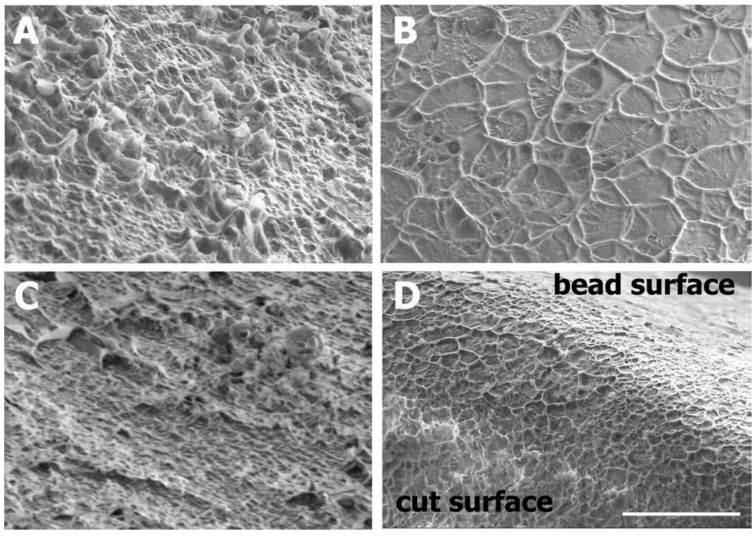
Cryo-SEM of alginate (Sigma A0682) beads showing external bead surfaces for (**A**) a 2% alginate extrusion bead, (**B**) a 2% alginate emulsion bead and (**C**) a 5% alginate emulsion bead, as well as (**D**), a 5% alginate bead cut in half, with the cut surface facing the camera. The scale is the same for all images. Adapted with permission from [43], Wiley Online Library, 2012.

SEM provides accurate information on the topography of nanocarriers with direct visualization. Typically, samples are dried, seated on a sample holder and coated by a metal with high electric conductivity, such as gold, using a sputter coating process. The sample surface is scanned with a focused beam of electrons, and the secondary electrons emitted from its surface are recorded [40]. Nanocarriers scanned by SEM should ideally be able to withstand the coating material, electron beam and vacuum without substantial change. Modified SEM techniques have been introduced, in which sample drying is not necessary. For example, wet SEM allows the analysis of hydrated samples without fixation, coating or drying [41,42]. Cryo-SEM is another modified technique that requires sample freezing, Figure 2 [43].

#### 2.3.2. Transmission Electron Microscopy

**Figure 3:**
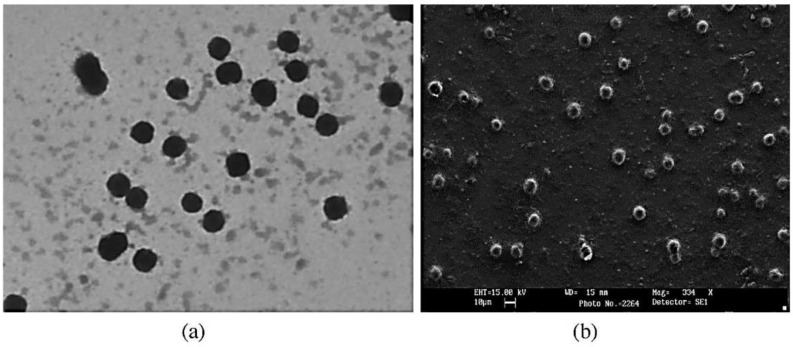
(**a**) TEM and (**b**) SEM images of lyophilized DLX-NLC. These micrographs revealed the nanoparticulate (80.17–127.73 nm) and spherical nature of DLX-NLC. Adapted with permission from [47], Elsevier, 2014.

TEM supplies information about the morphology of nanocarriers through which electrons are passed. However, sample preparation for TEM is relatively complicated and takes too much time as samples should, ideally, be ultra-thin to allow electron transmittance [44]. The nanocarrier sample is mounted onto support films or grids and fixed using either a negative stain material, such as uranyl acetate or phosphotungstic acid, or plastic embedding. Alternatively, the sample is exposed to liquid nitrogen after inclusion in vitreous ice, also known as cryo-TEM [45]. Bio-polymeric samples frequently need staining by heavy metals to exert sufficient contrast to be identified. The internal structure of particles can also be investigated after the fixation, drying up and cutting of the sample [46]. A comparison of SEM and TEM imaging is demonstrated in Figure 3 [47].

## 3. Composition, Loading Efficiency and Mechanical Properties

One of the most challenging issues after the preparation of the nanocarriers, due to the tiny dimensions engaged, is the determination of the exact particle constitution and the allocation of the active ingredients within the particles [20]. X-ray photoelectron spectroscopy can be used to examine the chemical constitution of the surface of the nanocarriers via elemental analysis, which indicates whether the drug is successfully encapsulated [48]. Additionally, Raman spectroscopy can give data on the surface properties of the molecules based on their respective vibrational transitions [49,50]. To detect the interactions between the polymers and crystals of an encapsulated compound, differential scanning calorimetry (DSC) is frequently required [51,52]. In addition, polymeric interactions can be investigated using infrared spectroscopy [53], while X-ray diffraction analysis may reveal the crystallinity status of nanocarrier components [54].

Entrapment efficiency (EE) is one of the leading variables for the characterization of nanocarriers. Two methods are often used for EE measurement: the direct and indirect methods [55]. In the indirect method, EE is measured by determining the concentration of the unentrapped drug in the supernatant layer after centrifugation (Equation (1)).

(1) $$\%\mathrm{EE}=\frac{\mathrm{Initial}\;\mathrm{amount}\;\mathrm{of}\;\mathrm{the}\;\mathrm{drug}-\mathrm{Free}\;\mathrm{unentrapped}\;\mathrm{drug}}{\mathrm{Initial}\;\mathrm{amount}\;\mathrm{of}\;\mathrm{the}\;\mathrm{drug}}\times 100$$

On the other hand, in the direct method, the concentration of the entrapped drug is directly measured inside the nanoparticles by the solubilization of nanoparticles in a suitable solvent, followed by filtration and drug analysis by the appropriate method (Equation (2)).

(2) $$\%\mathrm{EE}=\frac{\mathrm{Amount}\;\mathrm{of}\;\mathrm{entrapped}\;\mathrm{drug}}{\mathrm{Initial}\;\mathrm{amount}\;\mathrm{of}\;\mathrm{the}\;\mathrm{drug}}\times 100$$

Fresta et al. [55] obtained almost the same values of EE using the two different equations (<3%).

The certainty of drug analysis is the major obstacle in EE determination. Depending on the chemical structure of the drug, its loading efficiency can be quantitatively determined by UV spectrophotometry [56] and liquid chromatographic techniques such as high-performance liquid chromatography (HPLC) [55].

The mechanical properties, elasticity and hardness of nanocarriers are studied using nano-indentation, atomic force microscopy, micropipette aspiration, particle poking and optical tweezers [57,58].

## 4. In Vitro Drug Release

Drug release from nanocarriers is a prominent characteristic that predicts therapeutic efficiency and side effects, i.e., the in vivo behavior. Performing in vitro release testing helps in optimizing the formulation and evaluating batch-to-batch variation. In addition, it helps to ensure the compliance of the formulation with compendial regulations and the label claim [59,60]. Since one of the existing obstacles for determining drug release from nanocarriers is the lack of a regulatory standard, great care was taken in the creation of the appropriate tool to assess the in vitro release of nanocarriers [59]. In principle, constructing an in vitro drug release profile involves incubating the nanocarriers with release media, then withdrawing samples at predetermined time points and determining the released drug concentration [61]. Table 1 shows examples of studies that tested the in vitro drug release from nanocarriers using different methods.

### 4.1. Dialysis Method

It is the most widely used in vitro drug release method as it is characterized by ease of set-up and possible time sampling. It involves a two-compartment system and a dialysis membrane with a molecular weight cut-off (MWCO) that is at least 100 times higher than that of the drug [69]. The drug diffusion from the nanocarrier compartment to the other is quantified. This technique has been applied to a variety of nanocarriers such as nanospheres [82], liposomes [69] and nanoemulsions [67]. The dialysis method is described as ‘regular’ [83], ‘reverse’ [69] or ‘side-by-side’ [82] according to the set-up and volume of the donor and receiver compartments. Despite its simplicity, the dialysis method fails to practically mimic the drug release profile as the analyzed drug concentrations reflect not only its release from the nanocarrier system but also its diffusion through the dialysis membrane. Thus, proper selection of the dialysis membrane specifications, such as the MWCO, charge and binding affinity, as well as the interpretation of the data with reliable mathematical models, could improve the reliability of results [84,85].

### 4.2. Sample and Separation Method

This method involves the effective separation of the nanocarriers from the release medium using syringe filters [73,74,86], centrifugation, ultracentrifugation or ultra-filtration [75,87,88]. Following sampling, a similar quantity of fresh medium is placed on the release medium to maintain sink condition. This method can be tailored by adjusting the set-up through changing the size of the container, method of agitation and sampling procedures. Widely reported set-ups incorporate USP I (basket), USP II (paddle) and vials. Agitation of release media is crucial here to avoid the aggregation of nanocarriers [89]. Agitation is accomplished via USP I and USP II apparatuses, magnetic stirrers or orbital shakers. Challenges facing this technique in the lab are the aggregation of the nanocarriers, blocking filters during sampling and drug adsorption to the filter [89]. Although sink conditions are recommended, non-sink situations are described to be preferable with poorly soluble drugs [90].

### 4.3. Continuous Flow Method

USP IV apparatus or a modified version is used [91]. Nanocarriers are exposed to a minor volume of pumped release media, which is afterward crossed a filter and analyzed. This technique is suitable for nanocarriers administered via subcutaneous or intramuscular routes as they become relatively confined within the administration site with limited exposure to biological fluids. However, set-up complications, filter clogging, drug adsorption and difficulty in maintaining a constant flow rate lead to variation in results [78].

### 4.4. Dynamic Dissolution Method

This method is direct and fast as it removes the need for sample separation [85,92]. Moreno-Bautista and Tam [85] reported the use of a dialysis membrane and drug-selective electrode to provide a robust release quantification of hydrophilic drugs, while Mora et al. [93] developed an approach based on voltametric electrodes to monitor the release of chemotherapeutic agents from liposomes. However, this technique lacks sensitivity and consistency of responsiveness. Other non-electrochemical methods, such as calorimetry [94], turbidimetry [95] and laser diffraction [96], are used as well.

### 4.5. Microdialysis Method

In this approach, microdialysis probes are placed into the dissolution containers and continually perfused with their release media using an internal tube. The media steams back among the internal tube and the external dialyzing film. The released drug in the media is afterward analyzed. This method has been effectively used for in vitro release studies of nanoemulsions, nanospheres and nanocapsules [97]. Fortunately, this technique does not disturb the balance between the encapsulated and free drug [87].

Mathematical modeling of drug release is essential to elucidate release mechanisms and, hence, optimize the formulation [98]. Usually, the release profile of nanocarriers includes four stages: a primary eruption stage due to the liberation of drug molecules next to the surface, a generalization stage where the drug is liberated with a comparatively fast rate, a slow-release stage and a final liberation stage [99]. Elements affecting the release kinetics out of nanocarriers include drug positioning, solubility and diffusion through the nanocarrier matrix. The Weibull, reciprocal powered time and three-parameter models are examples of mathematical models suitable for release profiling of nanocarriers involving dissolution and diffusion mechanisms [100,101].

In vitro in vivo correlation (IVIVC) is essential to identify the relationship between in vitro testing and in vivo blood plasma drug concentration of a DDS [102]. However, generation of a reasonable IVIVC is challenging as it requires standardized reference materials, optimized in vitro examination, determination of the nanocarrier biodistribution, control of the pharmacokinetics, examination of the nanocarriers’ transfer through several compartmental borders and improvement of suitable risk–benefit modeling [103]. On the other hand, Cao et al. [104] obtained an excellent IVIVC linear correlation between the in vitro dissolution of the prepared mesoporous silica nanoparticles encapsulating silybin meglumine and in vivo absorption for 72 h. Singh and Pai [105] reported an optimized nanoparticulate (NP) DDS using Eudragit RL 100 for *trans*-resveratrol (*t*-RVT). Efforts were made to relate the in vivo drug plasma level, achieved for the enhanced formulation of *t*-RVT NPs, pure drug and marketed formulation, and in vitro drug release data and an excellent IVIVC were established.

A correlation of this type is generally linear, representing a point-to-point relationship and is considered to be the most informative method.

## 5. Stability Studies

Ideally, nanocarriers should be stable, resist aggregation and retain the drug until reaching the target site. Stability issues may compromise the in vivo efficacy. Therefore, stability assessment of nanocarriers is an essential characterization step. There are several techniques for predicting the stability of nanocarriers, physically, chemically and in physiological surroundings, depending on the nature of the nanocarriers.

### 5.1. Stability Studies for Vesicular Nanocarriers

Oxidation of phospholipids, low zeta potential, incorrect charge allocation and aggregation as a result of Ostwald’s ripening are the most commonly encountered physicochemical stability issues for vesicular nanocarriers such as liposomes [106]. Consequently, aggregation, bilayer fusion and drug leakage may occur. To investigate long-term drug leakage upon storage and whilst in the general circulation, EE and drug release are evaluated [107]. Additionally, the storage temperature of nanocarrier dispersions should be controlled due to its prominent effect on drug release. Chemical instability is another problem observed. As the vesicles are consistently in the vicinity of watery media, the phospholipids (phosphatidylcholine) available in the vesicles prioritize hydrolysis to lyso-derivatives (lysophosphatidylcholine), which is recognized to be harmful to the integrity of vesicles and can be detected by chromatographic methods coupled with an evaporative light scattering detector [108,109].

### 5.2. Physical Stability of Self-Assembled Nanocarrier Systems

The self-assembled micelles include polymeric and surfactant micelle systems. The concentration at which self-assembled micelles associate, also known as critical micelle concentration (CMC), offers a quantitative evaluation of the physical stability of nanocarriers. It was previously reported that a relatively low CMC denotes increased stability [110]. The ultra-low-CMC micelles utilize a sharp polarity contrast between a super hydrophilic zwitter ionic polymeric field and a super hydrophobic lipid field. They can stabilize the hydrophobic drugs in diluted conditions, as well as in serum, where conventional micelles failed. CMC can be measured using conductance surface-tension chromatography, fluorescent probes and scattering of light [111]. The application of these techniques is appropriate for micelles and self-assembled nanocarriers, the formation of which is affected by ingredient concentrations.

### 5.3. Thermal Stability of Polymeric Nanocarriers

Low critical solution temperature (LCST) is the temperature at which a thermosensitive polymer undergoes phase transition. Under LCST, the polymer is amphiphilic, and the drug stays encapsulated, while, above LCST, the polymer becomes hydrophobic, releasing the drug [112]. LCST is a measure of the thermal stability of nanocarriers. Filippov et al. demonstrated that the LCST of poly(N-isopropylacrylamide-co-maleic acid) copolymer increased from 31 to 45 °C with increasing maleic anhydride content and molecular weight [113]. Conversely, Na and Bae confirmed that the LCST of Pluronics and poly(N-isopropylacrylamide) decreased upon mixing with saccharides [114].

### 5.4. Stability of Nanocarrier Suspensions and Nanoemulsions

Assuming that the nanocarriers are under a certain size range, diversions from the mean size might be indicative of dissociation or aggregation. Turbidity changes of the nanocarrier suspension may result from instability as well. The cloudiness of a sulfonamide-comprising nanocarrier suspension was determined at various pH values. At pH 7 or higher, the nanocarriers showed a constant particle size and turbidity, both of which increased upon lowering the pH below 7. This behavior indicates pH-induced agglomeration due to the hydrophobic association of the deionized polymer [115].

Nanoemulsions are thermodynamically undesired arrangements; due to the higher free energy of the emulsified form compared to that of the separated oil and water phases, they are liable to deteriorate over time. Hence, thermodynamic stability tests for flocculation, gravitational separation, Ostwald ripening, coalescence or phase inversion are crucial to determine the nanoemulsions’ stability [116].

### 5.5. Stability of Nanocarriers in Biological Matrices

When nanoformulations are exposed to complex biological matrices, such as blood [117,118,119,120], cerebrospinal fluid (CSF) [121], tears, aqueous humor [122], vitreous humor [123] and synovial fluid [124,125], their physicochemical characteristics can change dramatically [27]. The potential impact of the biological environment on nanoformulations includes surface coverage of most nanoparticles by a complex multilayer of proteins, called the “protein corona”. A remarkable change in their physicochemical characteristics may take place upon contacting the biological matrix, altering their surface properties, potentially generating an immune response and modifying fate, toxicity and targeting [126,127].

To comprehend how nanosurfaces and their interactions behave in biological systems, proper analytical tools are required. However, the small size, variable composition and surface chemistry of nanoparticles and extremely complex nature of biological matrices make their detection and characterization difficult. The adsorbed proteins on nanoparticles’ surfaces can be directly or indirectly detected.

Direct approaches, such as spectrometry (e.g., MS, NMR and FT-IR), circular dichroism, gel permeation chromatography (GPC) and microscopy (e.g., AFM, TEM), are used to examine the proteins that are adsorbed on the nanoparticle surface. While the adsorbed protein is evaluated indirectly by evaluating changes in nanoparticles’ properties, such as size and aggregation, surface charge, density, mass, absorbance and Förster resonance energy transfer (FRET), the parameters measured are then correlated with the amount of protein adsorbed. In both approaches, the techniques allow for detection of the adsorbed protein in situ, while other techniques require the removal of unbound proteins before measurements [27].

These techniques were discussed in detail by Carrillo-Carrion et al. [128].

Gel permeation chromatography and Förster resonance energy transfer are most frequently used to predict the stability of nanocarriers in physiological fluids. In GPC, the self-assembled nanocarriers are separated from their degradation components [129]. Although this approach is direct, interactions among the column beads and nanocarriers might affect the result of the analysis.

FRET involves the coupling of fluorophores to nanocarriers, where the resonant transfer of excitation energy from fluorophores (donor) to nanocarriers (acceptor) occurs and is monitored [130]. For example, the serum stability of polyethylene glycol–polyvinyl pyrrolidone polymeric micelles is lower than polyester-based micelles, as revealed by the FRET technique [131].

## 6. Permeability Assessment

In order to identify the in vivo behavior of nanocarriers, permeability studies are employed to guarantee the therapeutic outcomes of the nanocarriers and also to help understand how the morphology of the nanocarriers may affect their bioavailability [132].

Different permeability evaluation techniques can be used such as ex vivo, in vivo, in situ organ perfusion and cell culture-based models (Table 2).

### 6.1. Ex Vivo Models

In this model, the desired organ can be isolated and perfused with the nanocarrier formulation under study. Everted or non-everted gut sac models are usually used to examine the absorption dynamics of drug-loaded oral nanocarriers with high duplicability [133,137]. The effect of different excipients on the bioavailability of nanocarriers can be evaluated as well. These excipients may improve solubility, alter intestinal permeability or interfere with enzymes [149]. In the everted gut sac model, the jejunum, duodenum or ileum are removed, divided into 5–6 cm segments, rinsed and everted on a glass rod. One extremity of the gut is clamped and filled by Krebs solution at 37 °C. The other end is tied and transferred to an incubation flask containing the nanocarrier in oxygenated media at 37 °C [150]. Then, samples are withdrawn at different time intervals. Several factors dictate the permeability results such as animal factors (species, age, disease state, diet), gut segment factors (duodenum, jejunum, ileum, colon) and test conditions (pH, aeration). Despite its sensitivity, the application of this model is limited by the short-term intestinal viability, loss of enzymatic activity, constrained sampling [151], eversion-induced morphological damage and lack of sink conditions due to the small size of the receiver compartment [152]. In the non-everted gut sac model, the small intestine is cut into segments, each filled with the nanocarrier suspension, tied on both ends and placed into Ringer’s solution. The samples are withdrawn for analysis from outside the sac, and the entire medium is replaced with fresh medium at predetermined time intervals. This approach is simpler, demands a smaller suspension volume and allows successive collection of samples from the serosal side with less intestinal morphological damage [153].

Ex vivo models are generally inadequate for testing nanocarriers with sustained release profiles due to the rapid loss of viability of the intestinal segment that can be maintained for only two hours. As for oral digestion, enzymes and bile salts are not defined; these models are not ideal for oral bioavailability assessment, resulting in inadequate correlation with in vivo profiles, especially for liposomes that have great digestive accountability.

### 6.2. In Vivo Methods

The prediction of the in vivo performance of nanocarriers based only upon their physicochemical properties and in vitro assessment may be misleading. This is because there are some biological considerations affecting drug bioavailability such as efflux transporters and metabolic enzymes. Identifying oral bioavailability of nanocarriers is indispensable as it reflects their pharmacological efficacy. It can be assessed via analyzing drug plasma concentrations after oral administration. Non-human primates are the most predictive but expensive experimental animals used in bioavailability testing. Although rodents have a lower correlation to human data [154], they are widely used [155,156]. Rabbits were also utilized in several studies [157]. Briefly, nanocarriers are administered to fasting animals by oral gavage. Blood samples are collected over a time interval and analyzed [158]. In mice, the volume of the administered dose should not exceed 350 µL to avoid reflux into the esophagus [159].

**Figure 4:**
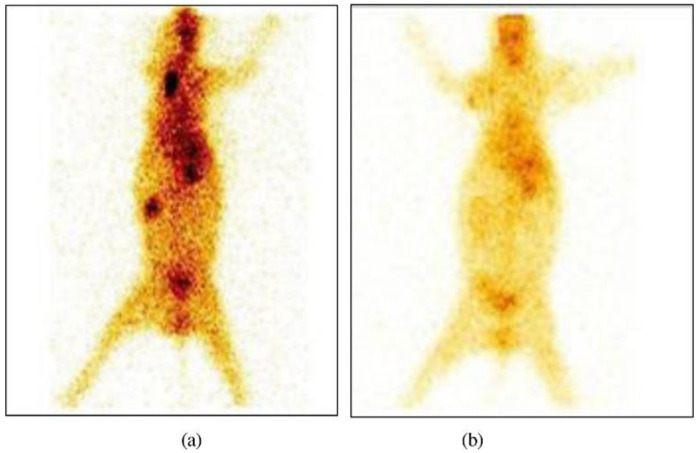
Gamma scintigraphy images after intranasal administration (6 h) of (**a**) DLX-NLC suspension, (**b**) DLX solution. These images show the localization of DLX in different organs, including brain of rabbit. DLX-NLC exhibited better localization than DLX. Adapted with permission from [47], Elsevier, 2014.

Alternatively, in vivo imaging techniques are employed to monitor the released drug biodistribution. Gamma scintigraphy, magnetic resonance imaging (MRI), single-photon computed tomography (SPECT), positron emission tomography (PET) and magnetic marker monitoring are examples [160]. In an investigation by Alam et al. [47], gamma scintigraphy was used to investigate the biodistribution and pharmacokinetics of duloxetine-loaded nanostructured lipid carrier (DLX-NLC) for nose-to-brain distribution. The drug was labeled by technetium radionuclide (^99m^Tc). The nanocarrier was intranasally administered to rats, and plasma samples were collected and analyzed using a gamma scintillation counter, while radioactivity in organs was determined by a shielded well-type gamma scintillation counter. For the whole-body gamma imaging study, rabbits were used, and localized radiation was envisioned using a single-photon emission computerized tomography (SPECT) gamma camera, Figure 4 [47]. The short half-life (6 h) and the safe radiation emission profile (radiation energy 140 keV) render ^99m^Tc an ideal radiotracer [161].

### 6.3. In Situ Organ Perfusion Models

Various transport mechanisms can be assessed by a non-evasive method, namely in situ perfusion. In this model, the animal is anesthetized, and a segment of the small intestine is cannulated and perfused with a solution containing a known concentration of the nanocarrier. The extent of drug absorption can be determined by comparing the drug concentration in the donor compartment with that in the solution exiting the intestine. The drug can be absorbed paracellularly through the intercellular clefts or transcellularly by permeating the apical membrane of the intestinal cells [162]. Singh et al. [163] utilized this model to verify whether the oral absorption of carvedilol could be enhanced by loading into solid, self-nanoemulsifying drug delivery arrangements. Improvement of carvedilol absorption was attributed to mechanisms of lymphatic transport and suppression of the P-glycoprotein efflux pump [163].

Compared to other models, this model allows the assessment of drug absorption directly. Moreover, it largely mimics the conditions in vivo in which integrated blood flow, nerves and clearance and expression capabilities of transporters and enzymes are maintained [164]. However, anesthesia and surgical handling of the gut may affect the gut blood supply and the absorption rate. Although the transmission assessment relies on drug loss in the perfusate, this does not entirely indicate the absorption rate as some drugs undergo pre-systemic metabolism [165].

### 6.4. Cell Culture-Based Models

Cell culture techniques are useful for assessing the permeability, cytotoxicity and targeting efficiency of nanocarriers (Table 2). Different culture models are utilized to evaluate the intestinal permeability of drugs [166]. These models exclude the interspecies variations in animal models, exhibit longer viability compared to ex vivo models and offer realistic permeability and transport data [166,167]. Diverse cell lines are grown on a semi-porous filter to make monolayers that, operationally, look like the intestinal epithelium with hindrance characteristics (tight junctions and microvilli). The monolayer is oriented in the dispersion machine containing apical and basolateral chambers that portray the mucosal and serosal layers of the gut, respectively [166].

Caco-2 cells are the most frequently used cultures for assessing the permeability of nanocarriers. As they originate from human colonic adenocarcinoma, they have multiple morphological and functional characteristics in common with human enterocytes and undergo differentiation in 21 days to form a confluent monolayer [168]. They exhibit brush boundary (microvilli) on the apical layers and express several transporters and metabolic enzymes that typically occur in the small intestine [169]. Therefore, Caco-2 cells are considered ideal for estimating approaches of drug transfer and permeation. In order to shorten the confluency time, an accelerated Caco-2 model was optimized. It only needs six days to forming a monolayer, which has better cost-effectiveness than the conventional Caco-2 model [170].

## 7. Challenges and Limitations of Nanocarrier Characterization

**Figure 5:**
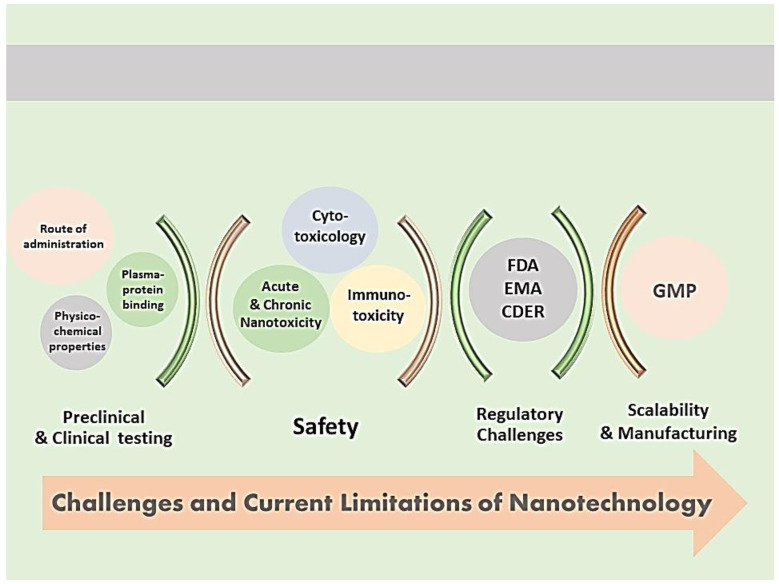
Pharmaceutical nanotechnology challenges and current Limitations. FDA—Food and Drug Administration; EMA—European Medicines Agency; CDER—Center for Drug Evaluation and Research; GMP—Good Manufacturing Practices.

Drug-loaded nanocarriers face multiple challenges for researchers and regulatory agencies (Figure 5). In order to overcome these challenges, robust characterization methods, scalable optimization approaches, safety guidelines and stability maintenance are needed [118,171,172].

### 7.1. Correlation of Preclinical Characterization to Clinical Testing

Currently, many nanocarriers are designed to function as tools for targeting, therapeutic purpose, imaging and controlled DDS. Their physicochemical properties are greatly influenced by the physiological environment; thus, their preclinical characterization becomes complicated. Developed reproducible standards to improve nanomaterials’ quality assessment are crucial for developing in vitro and in vivo models representing the clinical case. Regulatory organizations, the National Cancer Institute’s Nanotechnology Characterization Laboratory (NCL), Food and Drug Administration (FDA) and the National Institute of Standards and Technology (NIST), endeavor to develop and validate standardized characterization protocols for nanocarriers which are routinely revised and updated to include a broader range of nanotherapeutics.

Some difficulties may hamper the development of principles for depiction and the following clinical utilization of nanocarriers. Surfactants incorporated in nanocarrier formulations to promote dispersion usually interfere with conventional characterization methods. Nanocarriers with cationic surfaces can permeate cellular membranes more than neutral or anionic nanocarriers. This is also true with impurities and contaminants adsorbed to nanocarrier surfaces [173]. The physicochemical characteristics of nanocarriers, such as charge and hydrodynamic diameter, are highly affected by physiological conditions such as temperature, pH and ionic strength. Binding to plasma proteins upon administration may alter the distribution and clearance of nanocarriers, and the drug release profile might get completely changed in physiological fluids [174]. Furthermore, variations of polydispersity due to the interaction with body fluids may lead to significant alteration of toxicity profile and biocompatibility [175].

The route of administration greatly affects characterization methods as well. Intravenously injectable nanocarrier systems require fewer characterization protocols than nanocarrier complex formulations intended for oral, nasal or topical administration [176]. Therefore, it is essential to consider any possible interfering in vivo factors during in vitro characterization. To ensure data reliability, control samples with notable characteristics are usually incorporated in in vitro assays, together with analytical samples [177].

### 7.2. Safety Considerations

One of the most crucial issues involving the use of nanocarriers in DDSs is related mainly to their safety. Among the unique features possessed by the nanocarriers in DDSs, is the general concept of reduction in toxicity [178]. This can be attributed to the lower dose and better cellular uptake and targetability of nanocarrier DDS. However, the toxicity of several nanocarriers in the human body was investigated in different studies to ensure its safety [179,180,181,182]. The toxicity of nanocarriers was found to be related to various factors, including size, shape [183], surface charge [180], route of administration and drug dose [184]. The nano-scale dimensions of nanocarriers may lead to possible toxicity upon interaction with tissues and biological fluids [173]. It is recommended that cytotoxicity studies be conducted as an essential part of the in vivo characterization of nanocarriers. Acute toxicological interactions include hemolysis, inflammation, oxidative stress, impaired mitochondrial function, morphological changes, genotoxicity and skin and eye irritation, while chronic toxicities are even more complicated [185,186].

As conventional toxicity assessment methods designed for classical drugs are typically used, the resulting data are mostly inadequate. Cell culture models are frequently employed in acute nanotoxicity studies due to their simplicity and reasonable costs. However, they cannot be used to evaluate chronic toxicological outcomes due to limited cellular viability [187]. Furthermore, the toxicological consequences resulting from the repeated exposure of tissues to nanocarriers have not been adequately studied. Besides, many nanocarrier systems, such as peptides and nucleic acids, may provoke immunogenic reactions, leading to severe side effects and, more seriously, anaphylactic shock [188]. It was previously reported that nanocarriers are prone to stability problems upon long-term storage, which may influence their efficacy and toxicity. Large-scale manufacturing of nanocarriers necessitates a meticulous monitoring of exposure levels and any possible consequences. Adequate control of the nanocarrier manufacturing process will enhance the achievement of a favorable safety profile.

Novel toxicological approaches were previously reported such as particokinetics and multiparametric evaluation. To our knowledge, there is no globally standardized protocol for the toxicological evaluation of nanocarriers. According to some international, standard-setting bodies, the safety implications of nanocarriers should be considered, and size, surface charge and solubility can be used to predict the toxicity of nanocarriers [188]. It is preferable to wisely choose in vitro toxicity assays that show close similarity to in vivo conditions. Moreover, biodistribution studies may explain the reasons behind toxicological results [189].

### 7.3. Regulatory Challenges in Nanomedicine Development

The FDA and the European Medicines Agency (EMA) and other regulatory bodies, such as the Center for Drug Evaluation and Research (CDER), regulate the use of nanocarriers. Within the last 30 years, only 21 nanocarrier formulations have been approved [190]. Most of the approved nanocarriers are administered intravenously or orally and are mostly liposomes. They exhibit lower toxicity compared to the parent drug; yet, they mostly do not demonstrate improved efficacy [191]. It seems that transferring nanocarriers from bench to bedside remains challenging due to the lack of standardized and biorelevant guidelines for characterization and quality control. For instance, it is extremely difficult to establish a universal in vitro release method for nanocarriers [192].

There is a tremendous need to design and validate novel standardized protocols for the safety and characterization of nanocarriers, especially as there is not a long history of acceptance in literature. More importantly, regulatory bodies should differentially evaluate nanocarrier-based OTC cosmetic products, e.g., sunscreens from medical formulations [193].

### 7.4. Manufacturing Considerations

With the advancement of nanocarriers, comes the demand to develop scalable manufacturing techniques that apply good manufacturing practices for producing nanomedicines with optimized bioavailability and excretion profiles. Due to the polydispersity problem, it is challenging to achieve acceptable batch-to-batch reproducibility. Moreover, many large-scale process conditions need to be controlled such as polymer-to-drug ratios, lipid-to-drug ratios, solvents, temperature, pH, surfactants and sterility [145].

## 8. Integration of Artificial Intelligence (AI) with Nanotechnology

### 8.1. AI in Pharmaceutics and Drug Delivery

Lately, pharmaceutics and drug delivery have become more and more important in the pharmaceutical industry due to the extended time, increased cost and lower productivity of recent molecular commodities. However, even existing formulation development depends on classic trial and error experiments, which are time consuming, expensive and unpredictable. With the explosive growth of computing power and algorithms over the past decade, a new system called “computational pharmaceutics” is integrating big data, AI and multiscale modeling approaches into pharmaceutics, proposing significant potential change to the drug delivery paradigm. Nowadays, some actions are made to apply AI strategies to pharmaceutical product development, including pre-formulation physical and chemical properties and predicting activity, in vitro drug release, physical stability, in vivo pharmacokinetic parameters, drug distribution and in vivo–in vitro correlation [194].

In 2019, Run Han and colleagues applied machine learning methods to predict the physical stability of solid dispersion at 3 and 6 months [195]. Furthermore, in 2021, Hanlu Gao and colleagues examined the dissolution behavior of solid dispersion by machine learning. A random forest algorithm was used to generate a classification model to distinguish between two types of dissolution profile, “spring-and-parachute” and “maintain supersaturation”, with an accuracy of 85%, sensitivity of 86% and specificity of 85% in 5-fold cross-validation. The random forest algorithm was employed to create a regression model to predict the time-dependent total drug release with a mean absolute error of 7.78 in 5-fold cross-validation [194].

### 8.2. Applications of AI in the Development and Optimization of Nanocarriers

One current issue with drug delivery is its ability to target multiple receptors in the body, reducing the performance of a particular function [196]. Nanocarriers were found to have benefits in targeting drugs to specific cells or tissues, as they can be functionalized to target disease-specific cells, thus, preventing toxicity from being triggered in healthy cells [197]. Various properties of nanocarriers responsible for drug delivery are the size, shape, chemical composition and surface properties. However, preparing the optimal nanocarrier DDS is challenging [198]. The optimization of the nanocarrier–drug compatibility can be aided by AI and computational approaches to evaluate drug loading, drug retention and formulation stability [197].

The nanotechnology field is experiencing drastic differences in the technique and efficiency of experiments. A large number of laboratories currently use automated systems; however, the scaling-up of nanocarriers and AI-based databases has excellent promise in translation. The objective of integrating automation and AI proposes the chance to enhance targeted therapeutic nanocarriers for specific cell types and patients [199].

Molecular modeling investigations of nanocarrier DDSs have primarily focused on (i) evaluating nanocarrier formation and conformation, (ii) evaluating nanocarrier delivery and interactions, (iii) evaluating nanocarrier surface properties and (iv) nanocarrier adsorption on different surfaces [200].

There are a growing number of experimental tests to verify the properties of nanocarriers in vitro, in vivo and in disease areas. In 2020, Yuan He and colleagues used machine learning methods to predict nanocrystals [198]. The 910 particle size data and 310 PDI data covered high-pressure homogenization, wet ball-milling and anti-solvent sedimentation methods. The LightGBM models showed satisfactory performance of nanocrystals created by high-pressure homogenization and wet ball-milling methods [194].

In addition, cost-effective theoretical computational techniques can assist in avoiding the demand for numerous experiments with various drug combinations. Among these theoretical techniques, molecular dynamics and Monte Carlo simulations are the most widely used. In this way, simulations can clarify quantitative measurements that are difficult to obtain experimentally [196]. It is not easy to determine which nanocarrier scaffold is suitable for a particular application [201]. Additionally, each nanocarrier can be optimized to show the preferred behavior. In this regard, developing a repository that helps researchers to identify a suitable nanocarrier scaffold and their functional groups for specific drug encapsulation and release would represent a major advance. Efforts have been made to create a database repository of nanocarriers, where scientists can obtain 3D structures and physical and chemical properties, in “Collaboratory for Structural Nanobiology” [202]. Like the Protein Data Bank, this repository acts as a focal point to explain, organize and verify these structures, enabling correlations between the structures of nanocarriers and their toxicological, physical, chemical and biological data. Another repository that compiles the available literature related to various categories of nanocarriers, including metallic nanocarriers, polymers or dendrimers, is called the Nanomaterial Registry. eNanoMapper is a complete database that specifically focuses on the safety information of nanomaterials [203].

### 8.3. AI Problems in the Development and Optimization of Nanocarriers and Pharmaceuticals

The recent evolution of AI technologies has played a vital role in the rational design and optimization of nanocarriers and pharmaceuticals. The successful application of various AI techniques has decreased development time, assured product quality and promoted successful research and development of pharmaceuticals. However, while implementing machine learning algorithms, a familiar problem is data loss. The high cost of pharmaceutical trials and long research, preparation and optimization time cause this problem since large pharmaceutical companies usually strictly save their records and data. Moreover, there is no satisfaction anymore for people with the suitable performance of machine learning models but who also hope to understand their working mechanism. Interpretable machine learning methods can provide more in-depth insights into the development of pharmaceutical formulations.

In the future, greater integration of the pharmaceutical industry and AI techniques will provide more opportunities for research and development in the pharmaceutical field [194]. Additionally, a repository of nanocarriers in the 3D atom is still missing, which may also provide researchers with the opportunity for nanocarriers’ conjugation with various functional groups. Such a repository would allow researchers to smoothly assess the appropriate scaffold for performing molecular simulations [204]. Moreover, there is an urgent need for more researchers interested in handling and analyzing data [200].

## 9. Conclusions

Nanocarriers serve as revolutionary platforms to minimize toxicity, improve efficacy and achieve targetability of drugs. The development of hundreds of nanocarrier formulations over the last decades has introduced numerous in vitro and in vivo characterization techniques. Consequently, it has become more challenging to standardize the safety and manufacturing protocols that control the regulatory approval of those revolutionary systems.

Some considerations may facilitate the release of nanocarrier-based formulations to global markets. First, critical toxicity aspects related to the administration, biodistribution, metabolism and elimination of nanoformulations should be investigated. Second, refinement of the technical aspects that control the scalability, characterization and batch-to-batch reproducibility of nanocarriers is demanded. Finally, research groups, regulatory bodies and industrial sectors should cooperate to accelerate the development of safe, effective and stable nanocarrier DDS.

Although computational study and artificial intelligence will never substitute laboratory experiments, computational tools have an essential role in accelerating and improving target and drug discovery processes, as well as in discovering new, effective, computational tools for nanoparticle and DDS simulation, which are continuously evolving. Moreover, there is an urgent need for more researchers interested in handling and analyzing data.

## 10. Future Perspectives

Advances in surface technology of nanoparticles have allowed nanocarriers to engage applicants for future work involving targeted drug delivery. The utilization of nanotechnology in medicine has a great influence on human health in terms of diagnosis, prevention and treatment of illness. Numerous nanocarriers have been authorized for clinical use, and they are currently used to diagnose and/or treat several types of cancer. Additionally, there are different formulations, which are now in different stages of clinical trials.

Nanocarriers are intended to deliver drugs by different mechanisms: passive targeting, active targeting, solubilization and activated release. Nanocarriers increase therapeutic effectiveness, decrease the effective dose and decrease the danger of systemic, adverse effects. Key problems associated with the clinical development of nanocarriers were discussed, comprising biological difficulties, large-scale fabrication, biocompatibility and protection, intellectual activity, authority rules and whole cost efficiency compared to current therapies.

It is recommended to create an individualized therapeutic plan for nanomedicines, tailored according to the patient’s individual genetic and illness profiles. Scientists should consider reducing the complexity of nanocarriers and consider the final dosage form for human use so that the formulation is clinically applicable for therapeutic use.

Advances in nanotechnology have produced distinct opportunities to address challenges related to immunology and vaccine development, where the various architectures of nanoparticle systems modify novel platforms for the production of highly effective vaccines.

The accumulation of nanodrugs in unwanted tissues leads to toxicity problems. Therefore, in clinical studies, consideration should be given to the determination of the biological distribution of nanoparticles after systemic administration.

Lately, nanocarriers were widely investigated in vaccines against SARS-CoV-2 (that leads to COVID-19), with several effective late-stage clinical testing. Corporations, such as Moderna and BioNTech, utilize nanocarriers to encapsulate mRNA, which encrypts for a COVID-19 allergen. Since 30 November 2020, Moderna and BioNTech/Pfizer have met their main effectiveness cut-offs in phase III clinical tests and have claimed for urgent user authorization.

Recent results strongly suggest that nanomaterials have a bright future in the field of solid tumor therapy, which has led to the emergence of the need for more rational combinations of chemotherapeutic drugs and nanocarriers. Additionally, nanotargeted radiopharmaceutical moieties must be developed to diagnose and treat cancer. Safety assessments of nanomedicine will be an important topic to be developed in the future, in addition to its potential implications for the global economy; so, special regulations of nanotechnology are warranted.

## Figures and Tables

**Table 1:** In vitro drug release assessment techniques adopted for variable nanocarriers.

In Vitro Release Model	Subtype Model	Nanocarriers System	Reference
**Dialysis**	Regular Dialysis	Solid Lipid Nanoparticles	[62,63]
		Proniosomes	[64]
		Magnetic Nanoparticles	[65]
		Nanosponges	[66]
	Reverse Dialysis	Nanoemulsion	[67]
		Niosomes	[68]
		Liposomes	[69]
	Side-by-Side Dialysis	Nanospheres	[70]
		Nanostructured Lipid Nanoparticles	[71]
		Lipid Nanocapsules	[72]
**Sample** **and Separation**	Membrane Filters	Nanocrystals	[73]
		Mesoporous Nanoparticles	[74]
	Centrifugation	Chitosan Nanoparticles	[75]
	Ultracentrifugation	Liposomes	[76]
	Ultrafiltration	Chitosan Nanoparticles	[77]
		Liposomes	[76]
**Continuous Flow**		Nanoparticles Incorporated in Strip-Films	[78]
**Dynamic Dissolution** **Microdialysis**		Nanosuspension	[79]
		Nanofibers	[80]
		Nanoparticles	[81]

**Table 2:** Permeability assessment techniques of nanocarriers.

Permeability Assessment	Main Information	Nanocarrier Systems/Drugs (Technique or Part Used)	References
1. Ex vivo models	Examples of organs used: Intestine (everted gut sac and non-everted gut sac) Kidney Disadvantages: Not suitable for sustained-release nanoparticles due to the rapid loss of intestine segment viability (2 h) Not ideal for oral bioavailability, as bile salts and enzymes are not represented	Bilosomes/Acyclovir (Everted gut sac)	[133]
		2. Soy lecithin-chitosan hybrid nanoparticles/Raloxifene hydrochloride (Everted intestinal sac)	[134]
		3. Solid lipid nanoparticles/Linagliptin (Everted gut sac)	[135]
		4. Chitosan alginate nanoparticles/Furosemide (Non-everted gut sac)	[136]
		5. Single-shell nanoparticles/Iohexol (Kidney)	[137]
2. In vivo methods	Experimental animal models: Non-human primates: the most predictive, but expensive; Rodents: have a lower correlation to human data, but cheap, available and are widely used; Rabbits: could be used. Examples for in vivo imaging techniques: Gamma scintigraphy; Single-photon computed tomography (SPECT); Positron emission tomography (PET); Magnetic resonance imaging (MRI); Magnetic marker monitoring.	Polyester-based nanoparticles/Rifampicin (Bioimaging)	[138]
		2. Polymeric nanoparticles/Quetiapine (Gamma scintigraphy)	[139]
		3. Stabilized monoolein-based cubosomes/Paclitaxel (IVIS in vivo imaging system)	[140]
		4. Bubble-generating nano-lipid carriers/Doxorubicin (Ultrasound imaging)	[141]
		5. Zein nanoparticles/Thiamine conjugate (SPECT-CT imaging)	[142]
3. In situ organ perfusion models	Advantages: Allows the assessment of the drug absorption directly; Greatly simulates the in vivo conditions.	Solid lipid nanoparticles/Linagliptin (in situ intestine perfusion)	[135]
		2. Natural polysaccharide-cloaked lipidic nanocarriers/Curcumin (in situ intestine perfusion)	[143]
		3. Solid lipid nanoparticles/ (in situ intestine perfusion)	[144]
4. Cell culture-based models	Examples: Cell line/origin: Caco-2/Human colon adenocarcinoma; J774 macrophages; MCF-7/Human breast adenocarcinoma; HepG2/Hepatocellular carcinoma cells; MCF-7/breast cancer cells and L929/normal cell	Colloidal nano silver/extract of Eucalyptus Camaldulensis leaves (Caco-2/Human colon cancer)	[145]
		2. Oleuropein/Nanostructured lipid carriers (J774 murine macrophages)	[146]
		3. L-carnosine-coated magnetic nanoparticles (MCF-7/Human breast adenocarcinoma)	[65]
		4. Mesoporous silica nanoparticles/ruthenium complex and conjugated with folic acid (HepG2/Hepatocellular carcinoma cells)	[147]
		5. pH-sensitive biocompatible and multifunctional nanocarrier/Paclitaxel (MCF-7/breast cancer cells and L929/normal cell)	[148]
